# Frequency of and sex distribution in specific phobia subtypes in a treatment-seeking sample

**DOI:** 10.1192/bjo.2025.10767

**Published:** 2025-08-01

**Authors:** David Veale, Charles Beeson, Andriani Papageorgiou

**Affiliations:** Institute of Psychiatry, Psychology & Neuroscience, King’s College London, UK; South London and Maudsley NHS Foundation Trust, London, UK; Centre for Anxiety Disorders and Trauma, The Maudsley Hospital, London, UK

**Keywords:** Specific phobia of vomiting, emetophobia, prevalence, epidemiology, clinic sample

## Abstract

**Background:**

Specific phobias are common in the community, and much is known from epidemiological surveys about their subtypes and sex ratio.

**Aims:**

To determine the subtypes and sex ratio in a treatment-seeking sample of people with a specific phobia.

**Method:**

Patients with a specific phobia were identified by a retrospective search of clinical case records from patient notes in electronic health records at the South London and Maudsley NHS Foundation Trust (the largest secondary mental healthcare provider in Europe).

**Results:**

We identified 1017 patients over 5 years as having a specific phobia. The adult female to male sex frequency ratio for having any specific phobia was 3.9, with the ratio of specific phobia subtypes ranging from 2.4 (natural environment) to 8.2 (animal). The child female to male ratio of specific phobia subtypes ranged from 0.7 (natural environment) to 1.8 (other subtypes). Phobia of vomiting was the most common specific phobia presenting in both adults (*n* = 161, 17.8% of all specific phobias) and children (*n* = 26, 23.4%). In adults with a phobia of vomiting, the female to male ratio was 9.1 compared with 3.4 in all other specific phobias, and 4.2 versus 0.98 for children.

**Conclusions:**

There is a stark contrast between the apparent prevalence of phobia of vomiting in epidemiological surveys and being the most common presentation clinically. A very high female to male ratio in phobia of vomiting and animals in adults seeking treatment is also in contrast to findings in the community. This has implications for clinician training and public education.

A specific phobia is characterised by a marked and excessive fear or anxiety that occurs consistently when the subject is exposed to one or more specific objects or situations out of proportion to any actual danger posed. The phobic objects or situations are avoided or else endured with intense fear or anxiety. Symptoms persist for at least several months and are sufficiently severe to result in significant distress or significant impairment in functioning.^1,2^ In the fifth version of the Diagnostic and Statistical Manual of Mental Disorders, phobic stimuli are grouped into five subtypes: (a) animal (e.g. dogs, spiders, mice, insects), (b) blood–injury–injection (BII), (c) natural environment (e.g. heights, weather, darkness, water), (d) situational (e.g. enclosed spaces, flying on a plane, trains) and (e) ‘other’ (e.g. vomiting, choking or incontinence). However, subtyping has been discontinued in the 11th version of the International Classification of Diseases (ICD-11).^3^ Exposure therapy for specific phobia is the recommended treatment option, with evidence that it is effective over other types of therapy.^4,5^

## Prevalence and risk factors of specific phobia

The largest cross-national (25 countries) survey of specific phobia (*N* = 124 902) found a lifetime and 12-month prevalence of 7.4 and 5.5%, respectively, of specific phobia in adults, with a female to male ratio of 2.33 for 12-month prevalence.^6^ The highest frequency was for animals (3.8%), followed by BII (3.0%), high places (natural environment, 2.8%) and still water or weather events (natural environment, 2.3%). However, the ‘other’ subtype was not included in this study. The animal subtype is commonly evidenced as being the most common and with the highest female to male ratio,^7–10^ with dental phobia having a higher point prevalence relative to other phobia sub-types in one study conducted in The Netherlands.^11^

For children and adolescents, two studies reported a 12-month prevalence of specific phobia in children at between 7.9^12^ and 9.7%,^13^ with Benjet et al^12^ finding the natural environment subtype the most common. Female to male sex ratios for animal phobia were consistent in each study (2.3), but more males were affected by natural environment phobia in the study of Kim et al^13^ than in that of Benjet et al^12^ (ratio 0.9 *v*. 2.1). The ‘other’ subtype was not reported in either study.

Data show a higher prevalence of a specific phobia in people with a lower education level, who were formerly married and have depression.^14^ Female sex and comorbid major depressive disorder were the strongest risk factors for phobia-related worry in one study, as well as traumatic experiences involving significant others, number of chronic diseases and comorbid substance use disorders.^15^

## Community prevalence of secondary subtypes of specific phobia

Investigation into the prevalence of specific variants of each phobia category (for example, the different types of animal phobia) can provide insights into the specific treatment needs of populations. In community settings, snake and spider phobias are among the most common anxiety disorders.^16^ For natural environment phobia, heights and water phobias have been evidenced as having the highest lifetime prevalence in general population samples in The Netherlands^17^ and the USA.^8^ For situational phobia, Stinson and colleagues^8^ found that being in closed spaces was the most common subtype (3.2%), followed by flying (2.9%), being in a crowd (1.6%) and bus, car or train travel (0.7%).

Epidemiological data for the BII subtype are more widely researched. Stinson and colleagues^8^ found that going to the dentist was the most common subtype (2.4%), followed by seeing blood or receiving an injection (2.1%) and visiting or being in hospital (1.4%). The prevalence of the BII subtype may vary considerably by age group: a recent review indicates that needle fear and phobia were substantially more common in younger age groups – 20–30% in 20- to 40-year-olds – compared with <5% in older age groups.^18^

Other specific phobia subtypes, such as the specific phobia of vomiting (SPOV, or ‘emetophobia’), have fewer epidemiological data. SPOV has an apparent rare community prevalence, with females being disproportionately affected.^19^ Becker and colleagues^10^ found a prevalence of just 0.2% for SPOV in their survey (*n* = 2064), in which the 12-month prevalence of specific phobia in the community was 10%. Two small surveys of people with a SPOV suggested a high female to male ratio (range 8.3–32.3).^20,21^ Further epidemiological data on SPOV diagnosis prevalence are warranted.

## Prevalence of specific phobia in clinical settings

Conducting prevalence studies on specific phobia in treatment-seeking samples helps identify which phobia subtypes are the most common. This supports efficient resource allocation and highlights areas where clinicians may need additional training.

Two studies in child and adolescent treatment-seeking samples were identified. Strauss and Last^22^ (*N* = 38), found that animal (33.3%) and situational phobias (38%) were the most common subtype and BII the least (6.9%). Furthermore, although their sample consisted of participants recruited to a randomised controlled trial, Ollendick and colleagues^23^ (*N* = 100) found that the most common specific phobias were the animal subtype (54%), the natural environment (25%) and situational (13%). Moreover, that study did not include any of the ‘other’ subtypes and both studies above had small samples. It is therefore difficult to generalise about the nature of specific phobias that present clinically in children and adolescents. Comprehensive specific phobia data in a large treatment-seeking sample of children are therefore needed.

We identified three studies in adult treatment-seeking samples. Himle and colleagues^24^ investigated the sex ratios of specific phobias in 88 patient records (62 females, 26 males).^24^ Situational phobias (crowded spaces, driving, buses, bridges, elevators and heights) were found to be the most prevalent (52.3%), followed by the animal subtype (28.4%); vomit–choking phobia occurred in 9%. These authors found a low female to male ratio in situational phobias (1.3) and BII phobias (0.8), whereas all 25 of the participants identified as having an animal phobia were women. Seven out of the eight participants with vomit–choking phobia were women, but other forms of vomit phobia were not reported.

Goisman et al^25^ drew upon a sample of 115 adults with comorbid anxiety disorders and found that the ‘other’ subtype (44%) and a fear of heights (natural environment) (41%) were the most common. They state that phobias such as ‘flying’, ‘water’, ‘crowds’ and ‘loud noises’ were among phobias within the ‘other’ subtype, but their respective frequencies were not provided. Furthermore, SPOV prevalence was not mentioned and overall sample size was low.

Given the limited epidemiological data of SPOV and how this compares with other specific phobias in regard to aetiology and treatment needs,^19^ Meule and colleagues^26^ compared the characteristics of 70 in-patients with SPOV to 40 other people with other specific phobias in a clinic in Germany. Nearly eighty per cent of SPOV individuals were female. Furthermore, SPOV subjects were younger and had lower body weight, higher phobic anxiety and lower self-reported life satisfaction than in-patients with other specific phobias. This might suggest that SPOV is a more severe specific phobia subtype and thus may result in a high proportion of individuals requiring treatment, leading to a comparatively high prevalence of SPOV relative to other specific phobias in treatment-seeking samples. However, the study of Meule and colleagues focused only on in-patients (which is unusual for specific phobias) and had a relatively small sample size. Further research comparing SPOV with other specific phobia subtypes is therefore warranted in a larger treatment-seeking sample consisting of predominantly out-patients.

## The present study

Given the very limited specific phobia data on treatment-seeking samples, we investigated the frequency of various specific phobia subtypes in clinical sites within a National Health Service trust in London, UK – the South London and Maudsley NHS Foundation Trust (SLaM). Specifically, we aimed to carry out the following:

(a) report the frequency of different specific phobias, including the female to male sex ratio for each subtype, in children and adolescents (aged under 18 years old) and adults.

Furthermore, because of neglected SPOV epidemiological data and evidence of its potentially greater impairment than other specific phobias^19,26^ we aimed to

(b) report the prevalence of SPOV in children and adults, including the female to male sex ratio, compared with all other forms of specific phobia.

## Method

The present study consisted of a search of clinical case records to identify cases with a specific phobia using the Clinical Records Interactive Search (CRIS) system. CRIS de-identifies and structures electronic health records from SLaM, which is the largest secondary mental healthcare provider in Europe. It covers the four London boroughs of Southwark, Lambeth, Lewisham and Croydon, with a catchment area of 1.3 million residents, as well as offering tertiary national referral units. SLaM’s geographical catchment area is diverse, and is representative of London’s overall population in terms of sex, education, age and socioeconomic status.^27^

The system for recording clinical notes became electronic throughout the trust in 2006. This information was made accessible for research by the establishment of the CRIS tool – an anonymised copy of the database – in 2008. The system can be searched for specific demographic and clinical structured fields (e.g. numerical data, ICD-10 diagnosis) as well as words or phrases within the vast volumes of free text (e.g. assessments, session notes and discharge summaries).

### Procedure

#### Search terms and classification criteria

We used the following terms to search for specific phobias using CRIS: ‘phobia’ and ‘phobic’. The time frame 2011–2015 is arbitrary, because this was the period for which data were available when the search was conducted. Structured clinical and demographic categories including sex, date of birth, ethnicity, location, hospital status and clinical summaries were selected for return for each case. Age was recorded as that when the individual first presented.

The free text and diagnosis summary were reviewed by two of the co-authors for each case and returned in order to classify cases into their respective subtypes: ‘animals’, ‘blood and injury [BII]’, ‘natural environment’, ‘situational’ and ‘other’.

#### Data extraction

Data were first extracted from both primary mental healthcare records (Improving Access to Psychological Therapies [IAPT], now called Talking Therapies) and secondary (based at SLaM). Primary mental healthcare providers deal with people suffering from mild to moderate mental health problems, while those with more severe and enduring disorders are referred to secondary mental healthcare.

Information was searched for according to the patient’s referral date, including all patients who were diagnosed in SLaM and IAPT services with a specific phobia (F40.2), or where a potential first diagnosis of specific phobia (free text search) was present in clinical notes. Individuals were classified as having a specific phobia if, based on the information available, they experienced an unreasonable or irrational fear related to exposure to specific objects or situations, and where this fear could not be better accounted for by an alternative diagnosis.

Each of the returned cases was then searched for individually, in order to return all free text for each person (i.e. including text without the terms ‘phobia’ and ‘phobic’). This enabled the researcher to search for other potentially relevant terms, such as ‘fear’ and ‘phobias’, and thus extract more information regarding the context of the specific phobia, in addition to other details such as comorbidities and other episode problems at the time of first diagnosis or first potential diagnosis of a specific phobia.

### Eligibility criteria

#### Exclusion criteria

At both the classification and review stage, cases were excluded from further analysis in the following contexts:

- false positives such as ‘no specific phobia’, ‘panic and phobia booklet’, ‘phobia scale’ and ‘phobia questionnaire’;
- where phobia was used to refer to another person;
- where the presence of the specific phobia was queried, including where uncertainty was expressed regarding whether the phobia was diagnostic;
- where the phobia was referred to fleetingly – for example as part of a previous presentation – with no further illuminating context;
- ‘agoraphobia’ or ‘social phobia’, as well as phobias relating to eating, contamination or germs, body weight, attending school, leaving the house and public speaking.

Due to the relatively large numbers of cases that were returned with ambivalent or false positive information, we chose to limit our review to those that were returned using the search terms ‘phobia’ and ‘phobic’.

#### Inclusion criteria

We included cases of:

- any age;
- diagnosis made during the 2011–2015 time frame.

### Ethics

CRIS received ethical approval from the Oxfordshire Research Ethics Committee, and full details regarding its protocols are outlined in an open access article.^28^

## Results

### CRIS search results

**Fig. 1:**
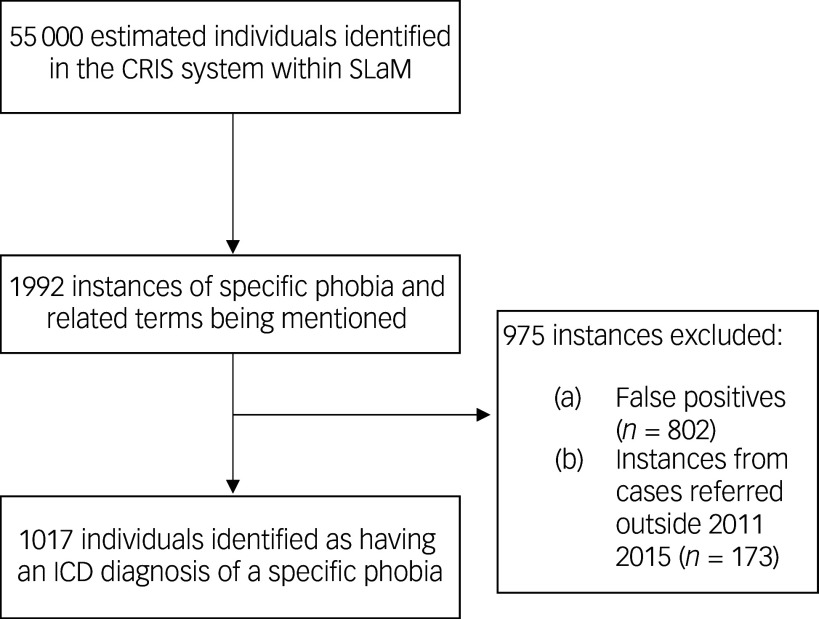
Data extraction flowchart of specific phobia cases identified on the Clinical Records Interactive Search system. SLaM, South London and Maudsley NHS Foundation Trust.

Figure 1 shows the total number of clinical records identified from the CRIS data search (an estimated 55 000 unique cases, on average, per annum) in the 2011–2015 period, and the final number of instances where specific phobia was deemed to be prevalent (*n* = 1017). There were approximately 40 000 individuals per annum with presentation to a service where the person was assessed (that is, had at least one assessment in hospital) and 25 000 per annum in psychological therapies services, totalling 65 000 instances of this. We were advised that there may have been some overlap between the two systems – for example, if someone is seen in both the hospital and the psychological therapies service – and hence the total number of patient episodes is estimated to be approximately 55 000 per annum.

The average number of specific phobia cases identified was 181 per annum for adults and 20 per annum for children, with an overall annual prevalence rate of 0.36% of all cases.

### Sample demographic and treatment characteristics

Table 1 shows the demographic characteristics and treatment status of the sample of individuals identified as having a specific phobia. There were 906 adults (mean age = 37.6 years) and 111 children (mean age = 11.0 years), and a significantly higher female to male ratio in adults compared with children. White was the most common ethnicity for both adults and children, and adults were statistically significantly more likely to have come from a White background than a Black and minority ethnic group compared with children and adolescents. There was also a significantly higher percentage of adult out-patients than in-patients compared with children and adolescents, as well as a higher percentage of locally as compared with nationally referred adult patients compared with children. ‘Locally’ contracts refers to the catchment area of 1.3 million people served by the hospital; ‘nationally’ refers to individual contracts from across England.

**Table 1:** Characteristics of adults, children and adolescents with a specific phobia

Characteristics	Adults (*n* = 906)	Children and adolescents (*n* = 111)	Test statistic (d.f.)	*P*-value	Effect size
Age (years, mean, s.d.)	37.6 (13.2)	11.0 (3.9)	*t*(498) = –45.8	<0.001	2.11
Sex (*n*, %)					
Female	722 (79.7)	63 (56.8)			
Male	184 (20.3)	48 (43.2)	*χ* ^2^ (1) = 29.5	<0.001	0.17
Ethnicity (*n*, %)					
White	624 (68.9)	61 (55.0)			
Asian	38 (4.2)	6 (5.4)			
Black	132 (14.6)	24 (21.6)			
Mixed	51 (5.6)	7 (6.3)			
Other	28 (3.1)	0	*χ* ^2^ (1) = 8.7	0.0032	0.090
Not specified	33 (3.6)	13 (11.7)			
Hospital status (*n*, %)					
In-patient	11 (1.2)	5 (4.5)			
Out-patient	895 (98.8)	106 (95.5)	–	0.023	0.070
Location (*n*, %)					
Local	883 (97.5)	71 (63.9)			
National	23 (2.5)	40 (36.1)	*χ* ^2^ (1) = 190.9	<0.001	0.44

The sample sizes reported in this table refer to the number of individuals with any specific phobia. Ethnicity chi-square test was performed on White versus Black and minority ethnic groups. Fisher’s exact test was used to compare hospital status between groups. Effect size for age comparison was calculated using Cohen’s *d*, whereas for all other comparisons Phi was used.

### Frequency of specific phobia subtypes

Table 2 shows the adult and child prevalence of different specific phobia subtypes and the corresponding female to male sex ratios. For children and adolescents (under 18 years), the most prevalent specific phobia subtype was ‘other’, followed by animals and BII; the least common were natural environment and situational. For adults, the most prevalent specific phobia subtype overall was ‘other’, followed by situational and animal; the least common were natural environment and phobia not specified (Table 2).

**Table 2:** Number of individuals with diagnosis of specific phobia subtypes in adults, children and adolescents

	Adults					Children and adolescents				
Phobia subtypes	Overall (*n*, %)	Age (years, mean, s.d.)	Female (*n*, %)	Male (*n*, %)	Ratio (female:male)	Overall (*n*, %)	Age (years, mean, s.d.)	Female (*n*, %)	Male (*n*, %)	Ratio (female:male)
Animals	203 (21)	37.7 (14.8)	181 (23)	22 (11)	8.2	33 (28)	11.2 (3.9)	17 (25)	16 (32)	1.1
Blood and injury	171 (18)	36.9 (13.4)	126 (16)	45 (23)	2.8	26 (22)	10.7 (3.9)	15 (23)	12 (24)	1.3
Natural environment	71 (7)	41.5 (14.3)	50 (6)	21 (11)	2.4	5 (4)	13.2 (2.8)	2 (3)	3 (6)	0.7
Situational	255 (26)	40.8 (12.2)	195 (25)	60 (31)	3.3	13 (11)	9.0 (4.1)	8 (12)	5 (10)	1.6
Other	265 (27)	34.1 (11.6)	223 (29)	42 (22)	5.3	39 (34)	11.6 (3.9)	25 (37)	14 (28)	1.8
Not specified	14 (1)	40.6 (11.4)	11 (1)	3 (2)	3.7	–	–	–	–	–
Total	979	37.6 (13.2)	786 (80.3)	193 (19.7)	4.1	117	11.1 (3.9)	67 (57.3)	50 (42.7)	1.3

Because an individual may present with more than one specific phobia type (for example, both animal and situational), the overall total frequency of specific phobias is higher than the total number of individuals identified in Table 1. If an individual presented with the same specific phobia subtype (e.g. animal) at different time points, this was counted only once in Table 2.

### Frequency sex ratio between specific phobia subtypes

In children and adolescents, the female to male ratio for all individuals with any specific phobia was 1.3, and 3.9 for adults. The highest sex ratio in the diagnosis of subtypes in children was for ‘other’ and situational, and least for the natural environment. The adult female to male sex ratio for the animal subtype was markedly higher in adults than for children. The environment subtype in children was the only specific phobia where more males were affected than females. The ‘other’ subtype constituted the highest proportion of all adult female specific phobia cases; the situational subtype was the most common specific phobia for adult male cases.

### Differences between SPOV and other specific phobias

Because there is some research comparing SPOV with other specific phobias^26^ and limited epidemiological data on SPOV, we compared the demographic characteristics of SPOV with all other specific phobias. Table 3 displays the differences in prevalence, sex ratio and demographic and treatment status characteristics between SPOV and other specific phobia subtypes in adults and children. Adults with SPOV presented at a statistically significantly younger age compared with all other specific phobias (mean difference age = 6.1 years, 95% CI: 4.1– 8.1, *t* = 6.1, *P* < 0.001). However, children with a phobia of vomiting were found to be significantly older at presentation than those with other specific phobias (mean difference age = 2.2 years, 95% CI: 0.5–3.9, *t* = 2.7, *P* = 0.01). Adults and children with a phobia of vomiting both had a markedly higher female to male ratio compared with other specific phobias. Adults and children with SPOV were also more likely to be in-patients than all other phobia subtypes (Table 3).

**Table 3:** Characteristics of specific phobia of vomiting (SPOV) compared with other subtypes in adults, children and adolescents

Characteristics	Adults					Children and adolescents				
	All other phobias	SPOV	Test statistic (d.f.)	*P*-value	Effect size	All other phobias	SPOV	Test statistic (d.f.)	*P*-value	Effect size
*N* (%)	745 (82.2)	161 (17.8)				85 (76.6)	26 (23.4)			
Age (years, mean, s.d.)	38.7 (13.4)	32.6 (11.2)	*t*(268) = 6.1	<0.001	0.47	10.6 (4)	12.8 (3.6)	*t*(2.7) = 45.1	0.01	0.57
Sex (*n*, %)										
Female	577 (77.4)	145 (90.1)	*χ* ^2^ (1) = 13.0	<0.001	0.12	42 (49.4)	21 (80.8)	*χ* ^2^ (1) = 7.98	0.0047	0.27
Male	168 (22.6)	16 (9.9)				43 (50.6)	5 (19.2)			
Female:male ratio	3.4	9.1				0.98	4.2			
Ethnicity (*n*, %)										
White	494 (66.3)	130 (80.7)	*χ* ^2^ (1) = 12.9	<0.001	0.12	40 (47.1)	21 (80.8)	*χ* ^2^ (1) = 9.14	0.0025	0.29
Asian	34 (4.6)	4 (2.5)				5 (5.9)	1 (3.8)			
Black	121 (16.2)	11 (6.8)				24 (28.2)	0			
Mixed	37 (5)	14 (8.7)				5 (5.9)	2 (7.7)			
Other	27 (3.6)	1 (0.6)				11 (12.9)	2 (7.7)			
Unspecified	32 (4.3)	1 (0.6)								
Hospital status (*n*, %)										
In-patient	3 (0.4)	8 (5)	–	<0.001	0.15	2 (2.4)	3 (11.5)	–	0.083	0.14
Out-patient	742 (99.6)	153 (95)				83 (97.6)	23 (88.5)			

These figures are based on the number of individuals with the specific phobia rather than numbers of diagnoses. Ethnicity chi-square test was performed on White versus Black and minority ethnic groups. Fisher’s exact test was used to compare hospital status between groups. Effect size for age comparisons is Cohen’s *d*, whereas for all other characteristics Phi was used.

### Frequency of secondary subtypes of specific phobia

The frequencies of each subtype for both adults and children are shown in Supplementary Tables 1–5. For adults, SPOV accounted for 17.8% of all cases of a specific phobia and 23.4% for children. SPOV accounted for 58.2% of all adult and child cases of the ‘other’ subtype (Supplementary Table 5). Subtypes could include more than one type. The most common specific phobia in the animal subtype for adults overall (*n* = 252) was spiders (*n* = 80), followed by mice and rats (*n* = 43) and dogs (*n* = 40) (Supplementary Table 1). In the BII subtype (*n* = 154) the most common was found to be needles and injections (*n* = 93), followed by blood (*n* = 54) (Supplementary Table 2). In the natural environment (*n* = 78) the most common phobia was heights (*n* = 59) (Supplementary Table 3), and in situational it was flying (*n* = 112) and confined spaces (*n* = 72) (Supplementary Table 4). The most common animal subtype for children (*n* = 37) was dogs (*n* = 9), followed by insects (*n* = 6) and spiders (*n* = 6) (Supplementary Table 1). For BII (*n* = 26) the most common subtype was needles and injections (*n* = 22) (Supplementary Table 2), for the natural environment (*n* = 5) it was wind and thunderstorms (Supplementary Table 3) and for situational (*n* = 17) it was public toilets (Supplementary Table 4).

## Discussion

To the best of our knowledge, this is the largest study conducted within a treatment-seeking sample of people with specific phobias. Our findings build on smaller studies conducted in clinical settings and provide further data for a phobia of vomiting.

We found a higher female to male sex ratio of having any specific phobia in adults compared with children (3.9 *v*. 1.3). In general, adult female to male ratios were markedly higher for all specific phobia subtypes (range 2.4–8.2) than child female to male ratios (range 0.7–1.8). Moreover, between the adult and child samples, the ‘other’ subtype had the highest frequency, which consisted predominantly of SPOV cases. Specifically, SPOV constituted 17.8 and 23.4% of all specific phobia cases for adults and children, respectively.

Notable differences between different specific phobia subtypes were found between adult and child samples. The only specific phobia subtype where more males were affected was environmental, the same as seen in one child and adolescent community study.^13^ Furthermore, a markedly higher adult female to male ratio for the animal subtype was found (8.2) compared with all other subtypes (range 2.4–5.3), but this ratio was markedly lower in children (1.1). For children, our finding that animal phobia was the most common specific phobia subtype (28% of all child-specific phobias) is broadly consistent with previous literature in clinical settings.^22,23^ However, situational phobia was markedly less relatively frequent in our child sample than in that of Strauss and Last,^22^ but consistent with Ollendick and colleagues.^23^ Furthermore, BII frequency in the present sample (23%) was markedly higher than that in Strauss and Last’s (6.9%)

Notably, the adult female to male ratio of 3.9 for any specific phobia in the present study is markedly higher compared with epidemiological survey estimates of 2.1–2.4,^6,8,9^ and may have been particularly biased by the high female to male ratio in phobia of vomiting and animals. This difference in sex ratio between community samples and the present study treatment-seeking sample might suggest that women are more likely than men to seek treatment for a specific phobia in the present study. The higher female to male ratio in this treatment-seeking sample is generally consistent with other anxiety disorders and depression.^29-31^

Adults with a phobia of animals also had double the female to male ratio (8.2) compared with specific phobias of the present study overall (3.9). This is also in contrast to epidemiological surveys that report a ratio of between 2.7 and 4.6 for animal phobias,^6,7,11^ and suggests that women are more likely to seek treatment than men for animal phobias such as spiders, rodents and domestic animals. This may be partly related to women experiencing a higher level of disgust sensitivity or having experienced more interference from spiders, rodents and domestic animals in the home environment.

For children and adolescents in the community, the figure for female to male ratio was 1.2^12^ or 1.5,^13^ similar to that of 1.3 in the present study. This suggests that, unlike adults, adolescents seeking treatment for a specific phobia are more representative of the community in terms of sex distribution and that they (or their parents) are equally likely to seek help for their specific phobia.

The high occurrence of a phobia of vomiting in this treatment-seeking sample highlights a need to detect SPOV cases in future epidemiological studies. We found a similar contrast in children, with the ‘other’ subtype and phobia of vomiting being the most common specific phobias found in our clinical setting, while the subtype with the highest prevalence in children in the community surveys was the natural environment.^12,13^

For a phobia of vomiting, there was also a high female to male ratio of 9.1 for adults: more than double that for all other phobias in our sample (3.4). Furthermore, the child female to male ratio for a phobia of vomiting was 4.2, compared with 0.98 for all other phobias. Our results are supported by other small surveys of a phobia of vomiting that found adult female to male ratios between 8.3^20^ and 32.3.^21^ This reflects Meule and colleagues’^26^ finding, where nearly 80% of SPOV in-patients were female. While our study could not explore the underlying reasons for this, it may be related to women experiencing a higher level of disgust sensitivity.^32,33^ There may be an evolutionary advantage in being more fearful of contagion or vomiting, especially during pregnancy or when looking after small children, who are at higher risk of vomiting. Men may have a more cavalier attitude to vomiting than women.

The relatively frequent occurrence of SPOV in this treatment-seeking sample contrasts with community epidemiological data in which SPOV was one of the least common subtypes.^8,10^ Impairment caused by phobia of vomiting may include school refusal; sleeping difficulties; postponing, terminating or avoiding a desired pregnancy; being unable to be left alone with young children; restricting food intake and becoming very underweight; leaving work or school if someone is potentially ill; or difficulty with travel or eating out.^20,21^ Thus, functional impairment associated with SPOV is high. Indeed, compared with individuals with other specific phobia subtypes, SPOV subjects were more likely to be in-patients than out-patients and they presented at an earlier age, on average, to services within the hospital. Additionally, Meule and colleagues^26^ found that SPOV in-patients had a significantly lower body mass index than that for other specific phobia subtypes, potentially due to higher rates of restrictive eating. Taken together, these findings suggest that SPOV may be more functionally impairing than other forms of specific phobia, leading to an earlier presentation at clinical services and requiring higher-intensity care.

However, it is possible that not all phobia subtypes have been systematically assessed in large epidemiological studies, including the present study. This issue may particularly affect frequency estimates of SPOV compared with other phobia subtypes, because some people with a phobia of vomiting may be diagnosed with obsessive–compulsive disorder (OCD),^34^ hypochondriacal disorder^19^ or even anorexia nervosa.^35^ Hence, the prevalence data of SPOV may be inaccurate. It is also possible that the epidemiological surveys of specific phobias have either not been comprehensive (because they did not include the ‘other’ subtype) or there is a difference in the way that clinicians diagnose a phobia of vomiting compared with the structured clinical interviews and questionnaires used in epidemiological surveys. There may need to be clearer guidelines or agreement about the differential diagnosis of OCD,^34^ anorexia^36^ and hypochondriacal disorder from a SPOV. This is not covered as a potential problem of boundaries with other disorders in ICD-11.^3^

### Research and clinical implications

The high frequency of SPOV in our study suggests that it should be better identified in future epidemiological studies. Moreover, the markedly high female to male ratio for SPOV in children and adults, and for animal phobia in adults, warrants further investigation. Research should explore underlying factors such as disgust sensitivity, functional impairment and other factors that may contribute to this disparity. This can help in understanding sex-specific vulnerabilities and the appropriate tailoring of interventions.

This also supports the need for SPOV to be treated as a priority among specific phobias in both teaching and research. Training programmes targeted towards in-patient and out-patient mental health clinicians could address topics such as recognising and treating SPOV, its potential impact on health (for example, reduced body weight, a reason for terminating a pregnancy or avoiding having children)^19,21^ and differentiating it from other conditions such as OCD and anorexia nervosa.

### Limitations

There are certain limitations to this study. It includes clinical data in which there is no structured diagnostic interview, and the records used may have been missing, contain inaccurate information or have been misinterpreted by the researchers. Nevertheless, a large database of clinical records can provide a helpful overview for understanding the type of presentation in a clinical setting that is not captured in a carefully controlled epidemiological survey. Furthermore, the findings of the present study may not be generalisable to other geographical regions.

In conclusion, there are significant differences between the types of specific phobia and sex ratio in the community compared with those in a clinical setting. Specifically, the presentation of SPOV is more common than suggested by community surveys. There is a very high female to male ratio in phobias of vomiting and animal subtypes. Future epidemiological surveys should identify people with the ‘other’ subtype and a phobia of vomiting. Teaching and research should focus on phobia of vomiting as being the most common type of phobia that presents in a clinical setting.

## Supporting information

Veale et al. supplementary material Veale et al. supplementary material

## Data Availability

The data that support the findings of this study are available from the corresponding author, D.V., upon reasonable request.
