# Supplementary material for: Frequency of and sex distribution in specific phobia subtypes in a treatment-seeking sample
Source: BJPsych Open. 2025 Aug 1;11(5):e164. doi: 10.1192/bjo.2025.10767 (PMC12344425; doi:10.1192/bjo.2025.10767)
Supplement: Veale et al. supplementary material [file S2056472425107679sup001.docx]

**Supplementary Materials**

**Note**: In all supplementary tables, all diagnoses at any time point for an individual are counted. This differs to Tables 2 and 3 in the main text, where the same diagnosis presenting at different time points is counted only once. For example, if someone presented with animal phobia in 2014 and again in 2015, this would be counted once in Tables 2 and 3 but twice in the supplementary tables. Consequently, the frequencies in the following supplementary tables are greater than or equal to those reported in the main text.

| **Supplementary Table 1**. *Numbers with a Diagnosis of Specific Phobia: Animal Subtype* | | |
| --- | --- | --- |
|  | Secondary Subtype | Specific Phobia |
| Adults (n= 252) | Arthropods n= 80 (31.7%)  Domestic Pets n= 47 (18.7%)  Rodents n= 43 (17.1%)  Insects n= 32 (12.7%)  All Animals n= 17 (6.7%)  Birds n= 17 (6.7%)  Fish n= 3 (1.2%)  Reptiles n= 9 (3.6%)  Wild Animals n= 4 (1.6%) | Spiders n= 80 (31.7%)  Dogs n= 40 (15.8%)  Cats n= 7 (2.8%)  Mice, rats n= 43 (17.1%)  Insects n= 19 (7.5%)  Slugs n= 2 (0.8%)  Worms n= 1 (0.4%)  Cockroaches n= 4 (1.6%)  Bees, wasps n= 2 (0.8%)  Butterflies, moths n= 4 (1.6%)  All Animals n= 15 (6.0%)  Fury Animals n= 1 (0.4%)  Small Animals n= 1 (0.4%)  Birds n= 17 (6.7%)  Fish n= 1 (0.4%)  Octopus n= 1 (0.4%)  Sharks n= 1 (0.4%)  Snakes n= 9 (3.6%)  Squirrels n=2 (0.8%)  Foxes n= 2 (0.8%) |
| Children (n=37) | Domestic Pets n= 12 (32.4%)  Insects n= 8 (21.7%)  Arthropods n= 6 (16.2%)  Birds n= 7 (18.9%)  All Animals n= 2 (5.4%)  Reptiles n= 1 (2.7%)  Rodents n=1 (2.7%) | Dogs n= 9 (24.4%)  Cats n= 3 (8.1%)  Insects n= 6 (16.2%)  Slugs n= 1 (2.7%)  Bees/ wasps n= 1 (2.7%)  Spiders n= 6 (16.2%)  Birds n= 7 (18.9%)  All Animals n= 2 (5.4%)  Snakes n= 1 (2.7%)  Mice, rats n= 1 (2.7%) |

*Note*. An individual may have had more than one animal SP (e.g. spider and snakes). All instances would be counted in this table.

| **Supplementary Table 2**. *Numbers with a Diagnosis of SP: Blood and Injury Subtype* | | |
| --- | --- | --- |
|  | Secondary Subtype | Specific Phobia |
| Adults *n*= 154 | Blood, Injury, Injection | Needles, injections n= 93 (60.4%)  Blood n= 54 (35.1%)  Blood, injury, injections n= 5 (3.20%)  Allergies n= 1 (0.65%)  Body Fluids n= 1 (0.65%) |
| Children *n*= 26 | Blood, Injury, Injection | Needles, injections n= 22 (84.6%)  Blood n= 4 (15.4%) |

*Note*. An individual may have had more than one BII SP (e.g. blood and allergies). All instances would be counted in this table.

| **Supplementary Table 3**. *Numbers with a Diagnosis of SP: Natural Environment Subtype* | | |
| --- | --- | --- |
|  | Secondary Subtype | Specific Phobia |
| Adults n= 78 | Natural Environment | Heights n= 59 (75.6%)  Darkness n= 7 (9.0%)  Wind, thunderstorms n= 3 (3.8%)  Water n= 3 (3.8%)  Grass, leaves n= 2 (2.6%)  Condensation n= 1 (1.3%)  Deep Holes n= 1 (1.3%)  Hills n= 1 (1.3%)  Sea n= 1 (1.3%) |
| Children n= 5 | Natural Environment | Wind, thunderstorms n= 4 (80%)  Heights n= 1 (20%) |
|  |  |  |
| *Note*. An individual may have had more than one natural environment SP (e.g. heights and darkness). All instances would be counted in this table. | | |
|  |  |  |

| **Supplementary Table 4**. Numbers with a Diagnosis of SP: Situational Subtype | | |
| --- | --- | --- |
|  | Secondary Subtype | Specific Phobia |
| Adults n= 344 | Travel n= 206 (59.9%)  Environment n= 27 (7.8%)  Claustrophobia n= 111 (32.3%) | Flying n= 112 (54.3%)  Tubes n= 28 (13.6%)  Transport, public transport n= 19 (9.2%)  Trains n= 16 (7.8%)  Driving n= 15 (7.3%)  Buses n= 6 (2.9%)  Cars n= 5 (2.4%)  Cycling n= 2 (1%)  Sailing n= 1 (0.5%)  Traffic n= 1 (0.5%)  Crossing roads n= 1 (0.5%)  Toilets, public toilets n= 7 (25.9%)  Roads, motorways n= 6 (22.2%)  Tunnels n= 3 (11.1%)  Bridges n= 2 (7.5%)  Crossing roads n= 1 (3.7%)  Hairdressers n= 1 (3.7%)  Open water n= 1 (3.7%)  Pavements, footpaths n= 1 (3.7%)  Stairs n= 1 (3.7%)  Thames Barrier n= 1 (3.7%)  Zoos n= 1 (3.7%)  Open spaces n= 1 (3.7%)  Scaffolding n= 1 (3.7%)  Confined spaces n= 72 (64.9%)  Lifts n= 36 (32.4%)  Escalators n= 3 (2.7%) |
| Children n= 17 | Claustrophobia n= 7 (41.2%)  Environment n= 6 (35.3%)  Travel n= 4 (23.5%) | Confined spaces n= 4 (57.1%)  Escalators n= 2 (28.6%)  Lifts n= 1 (14.3%)  Toilets, Public toilets n= 4 (66.6%)  Showers n= 1 (16.7%)  Stairs n= 1 (16.7%)  Flying n= 2 (50%)  Boats n= 1 (25%)  Transport, public transport n= 1 (25%) |

| *Note*. An individual may have had more than one natural environment SP (e.g. heights and darkness). All instances would be counted in this table.  **Supplementary Table 5**. *Numbers with a Diagnosis of Specific Phobia: Other Subtype in Adults and Children (the data are combined as there are too many other phobias to report)* | | |
| --- | --- | --- |
|  | Secondary Subtype | Specific Phobia |
| n= 380 | Other | Vomit n = 221 (58.2%)  Dentists n = 15 (3.9%)  Hospitals n = 15 (3.9%)  Choking n = 14 (3.7%)  Deaths n = 13 (3.4%)  Medical examinations n = 12 (3.2%)  Illness n = 9 (2.4%)  Labour/giving birth n = 7 (1.8%)  Incontinence n = 7 (1.8%)  Falling n = 5 (1.3%)  Medication n = 4 (1.1%)  Balloons n = 4 (1.1%)  Chewing noises n = 2 (0.5%)  Loud noises n = 2 (0.5%)  Doctors = 2 (0.5%)  Fire n = 2 (0.5%)  Glass/broken glass n = 2 (0.5%)  STDs n = 2 (0.5%)  Bumps/lumps n = 1 (0.26%)  Bright lights n = 1 (0.26%)  Butter n = 1 (0.26%)  Bites n = 1 (0.26%)  Burning buildings n = 1 (0.26%)  Buildings collapsing n = 1 (0.26%)  Being upside-down n = 1 (0.26%)  Children n = 1 (0.26%)  Cobwebs n = 1 (0.26%)  Colour yellow n = 1 (0.26%)  Drowning n = 1 (0.26%)  Fire alarms n = 1 (0.26%)  Fireworks n = 1 (0.26%)  Front door opening n = 1 (0.26%)  Fruit n = 1 (0.26%)  Ground collapsing n = 1 (0.26%)  Hoovers n = 1 (0.26%)  Knives n = 1 (0.26%)  Large surface areas n = 1 (0.26%)  Men n = 1 (0.26%)  Money n = 1 (0.26%)  Mud n = 1 (0.26%)  Nails n = 1 (0.26%)  Objects attached to strings n = 1 (0.26%)  Passing-out n = 1 (0.26%)  Photos n = 1 (0.26%)  Pollution n = 1 (0.26%)  Reading n = 1 (0.26%)  Rings on fingers n = 1 (0.26%)  Ropes n = 1 (0.26%)  Sexual intercourse n = 1 (0.26%)  Statues n = 1 (0.26%)  Shaking hands n = 1 (0.26%)  Swallowing n = 1 (0.26%)  Trampolines/swings n = 1 (0.26%)  Vaginal insertion n = 1 (0.26%)  Violence n = 1 (0.26%)  Walking downstairs n = 1 (0.26%)  Wax n = 1 (0.26%)  Witches = 1 (0.26%)  Writing n = 1 (0.26%)  Women n = 1 (0.26%) |

*Note*. An individual may have had more than one SP in the ‘other’ category (e.g. vomit and choking). All instances would be counted in this table.
